# Crystal Structures of Lsm3, Lsm4 and Lsm5/6/7 from *Schizosaccharomyces pombe*

**DOI:** 10.1371/journal.pone.0036768

**Published:** 2012-05-17

**Authors:** Donghui Wu, Shimin Jiang, Matthew W. Bowler, Haiwei Song

**Affiliations:** 1 Institute of Molecular and Cell Biology, Singapore, Singapore; 2 Life Sciences Institute and School of Medicine, Zhejiang University, Hangzhou, China; 3 Structural Biology Group, European Synchrotron Radiation Facility, Grenoble, France; 4 Department of Biochemistry, National University of Singapore, Singapore, Singapore; MRC National Institute for Medical Research, United Kingdom

## Abstract

Sm-like (Lsm) proteins are ubiquitous and function in many aspects of RNA metabolism, including pre-mRNA splicing, nuclear RNA processing, mRNA decay and miRNA biogenesis. Here three crystal structures including Lsm3, Lsm4 and Lsm5/6/7 sub-complex from *S. pombe* are reported. These structures show that all the five individual Lsm subunits share a conserved Sm fold, and Lsm3, Lsm4, and Lsm5/6/7 form a heptamer, a trimer and a hexamer within the crystal lattice, respectively. Analytical ultracentrifugation indicates that Lsm3 and Lsm5/6/7 sub-complex exist in solution as a heptamer and a hexamer, respectively while Lsm4 undergoes a dynamic equilibrium between monomer and trimer in solution. RNA binding assays show that Lsm2/3 and Lsm5/6/7 bind to oligo(U) whereas no RNA binding is observed for Lsm3 and Lsm4. Analysis of the inter-subunit interactions in Lsm5/6/7 reveals the organization order among Lsm5, Lsm6 and Lsm7.

## Introduction

Sm and Sm-like (Lsm) proteins have been found in all three kingdoms of life: bacteria, archaea and eukaryotes. They are essential parts of ribonucleoprotein (RNP) complexes and are actively involved in various steps of RNA metabolism including pre-mRNA splicing, mRNA degradation, telomere replication, histone formation and translational control [1]–[3]. Members of this protein family are characterized by two closely spaced, conserved Sm motifs 1 and 2, which adopt a conserved Sm fold that consists of an N-terminal α helix followed by a twisted five-stranded β sheet. A common characteristic of Sm/Lsm proteins is their tendency to form a hepta- or hexameric ring structure. The seven prototypical Sm proteins B, D1, D2, D3, E, F and G form a hetero-heptameric ring structure bound to a common U rich stretch termed as the Sm site of the U1, U2, U3, U4 and U5 small nuclear RNAs (snRNAs), which are essential for pre-mRNA splicing [4]–[6]. In addition to the hetero-heptameric complex formed by the seven canonical Sm proteins, eight Lsm proteins (Lsm1–Lsm8) have been shown to constitute three heteromeric complexes, namely, Lsm2–8, Lsm1–7, Lsm2–7 [7]–[10]. The specific composition and architecture of each Lsm complex determines its cellular location, RNA target and function in RNA metabolism [11], [12].

The Lsm2–8 complex is localized in the nucleus where it directly binds and stabilizes the 3′-terminal poly(U) tract of U6 snRNA [13] and facilitates the assembly of U4–U6 di-snRNP and U4–U6•U5 tri-snRNP [7], [13], [14]. In addition to its role in pre-mRNA splicing, the Lsm2–8 complex is also involved in processing of various nuclear RNAs, including tRNAs, snoRNAs and ribosomal RNAs, as well as in decay of nuclear mRNAs [11]. Lsm2–8 proteins have been shown to physically associate with some splicing factors [15]. Consistently, mutations in the Lsm2–8 complex show defects in splicing [14].

The Lsm1–7 complex made of seven Lsm proteins, Lsm1 through Lsm7, is highly conserved in all eukaryotes [7]–[9]. In contrast to the nuclear localization of the Lsm2–8 complex, this complex is localized to the cytoplasm, associates with deadenylated mRNA and promotes decapping in the 5′-3′ mRNA decay pathway [16]. The Lsm1–7 complex physically interacts with several decay factors involved in the 5′-3′ decay pathway, including Dcp1/Dcp2, Pat1 and Xrn1 in the discrete cytoplasmic foci known as P-bodies [9], [15]. The Lsm1-7-Pat1 complex purified from *S. cerevisiae* shows intrinsic affinity for the 3′ end oligoadenylated mRNAs over polyadenylated mRNAs, thus protecting this end from decay by the exosome while activating decapping [17]. Moreover, the Lsm1–7 complex has a strong binding preference for deadenylated mRNAs carrying a U-tract at their 3′ terminal over those that do not [17]. There is evidence showing that the Lsm1–7 complex binds certain viral mRNAs with a 5′ poly(A) tract, thereby stabilizing these mRNAs by inhibiting both 3′-5′ and 5′-3′ decay [18]. In addition to its role in general mRNA decay, the Lsm1–7 complex is involved in histone mRNA decay [19], [20], uridylation-mediated mRNA decapping [21], [22] and microRNA (miRNA) biogenesis [23]–[27] by recognizing and binding to the 3′ poly(U) tract of these mRNAs.

In addition to Lsm1–7 and Lsm2–8, a third Lsm complex, consisting of Lsm2–7 proteins has been identified in *Saccharomyces cerecisiae*. Unlike Lsm1–7 and Lsm2–8 which are localized in the cytoplasm and nucleus, respectively, Lsm2–7 resides in nucleoli and associates with the small nucleolar RNA snR5 [10] that functions to guide site-specific pseudouridylation of rRNA, suggesting that this complex contributes to the biogenesis or function of specific snoRNAs.

In contrast to the 18 or more Sm/Lsm proteins identified in eukaryotes, bacteria and archaea contain only one or two Sm/Lsm proteins [2]. Crystallographic study of several bacterial and archaeal Sm/Lsm proteins show that they form an overall doughnut-shaped ring structure of a hexamer or a heptamer. Two faces termed as “helix” face and “loop” face are located at the opposite sides of the ring structures with the U-rich oligoribonucleotides bound at the “helix” face [28]–[30]. Based on crystal structures of SmB-SmD3 and SmD1–SmD2 hetrodimers, the seven Sm proteins have been proposed to form a heptameric ring around the Sm binding site of snRNAs [31]. Most recently, crystal structures of U1 snRNP and U4 snRNP core domain were reported. These two structures clearly reveal the hetero-heptameric ring organization formed by the seven Sm proteins in a clockwise order of B, D1, D2, F, E, G and D3 and the ring wraps around the Sm site of U1 snRNA and U4 snRNA [5], [6]. Notwithstanding the fact that the formation of the heptameric ring of seven Sm proteins requires the presence of each U snRNA, the Lsm2–8 complex has been shown to be stable in the absence of its cognate U6 snRNA, suggesting that this complex assembly is independent of RNA [13]. Consistent with this observation, the Lsm2–8 complex can be reconstituted in vitro by mixing the coexpressed and purified Lsm2/3, Lsm4/8 and Lsm5/6/7 sub-complexes [32]. The Lsm1–7 complex, which has Lsm2 to 7 in common with the Lsm2–8 complex and differs only in the seventh subunit (Lsm1 and Lsm8 respectively), can also be assembled in vitro without RNA by a combination of purified Lsm1, Lsm4, Lsm2/3 and Lsm5/6/7 sub-complexes [32]. Electron micrographs show that reconstituted Lsm1–7 and Lsm2–8 have a ring-like architecture and are similar to one another and to the native Sm/Lsm complexes, suggesting that the architectures of these two complexes follow the generic Sm/Lsm complex pattern [32]. Despite these advances on the in vitro assembly of Lsm1–7 and Lsm2–8 complexes, no crystal structure of either of these two complexes has been reported.

As the first step towards understanding the assembly of Lsm1–7 and its function, we have determined three crystal structures including Lsm3, the N-terminal region of Lsm4 and Lsm5/6/7 sub-complex from *S. pombe* (designated as SpLsm3, SpLsm4N and SpLsm5/6/7, respectively). These structures showed that all five individual SpLsm proteins (SpLsm3 to SpLsm7) adopt a common Sm fold. Structural data combined with analytical ultracentrifugation analysis clarified the oliogomeric states of SpLsm3, SpLsm4N, and SpLsm5/6/7. Surface plasmon resonance analysis in combination with fluorescence anisotropy analysis revealed that SpLsm2/3, and SpLsm5/6/7 bound to oligo(U) whereas no binding of oligo(U) was observed for SpLsm3 and SpLsm4N. The structure of Lsm5/6/7 revealed that Lsm5 bridges the interaction between Lsm6 and Lsm7.

## Results and Discussion

### Structural Determination

Structure determination of SpLsm2/3 was attempted at a resolution of 2.7 Å. To our surprise, only SpLsm3 was identified in the asymmetric unit (AU). One possibility is that SpLsm2 was lost during crystallization as the crystals were obtained from the heavily precipitated mother liquor. Consistent with this possibility, SDS-PAGE of the protein samples prepared from the thoroughly washed crystals showed that only SpLsm3 was identified in the crystals, thereby confirming that SpLsm2 was precipitated out during crystallization process (Data not shown). The structure of SpLsm3 was solved by single-wavelength anomalous dispersion (SAD) phasing method using a SeMet-substituted crystal. The final model has been refined to an R factor of 24.3% and R_free_ of 27.7% with good stereochemical geometry. Residues 1–8 in the N-terminal and residues 56–69 in the loop region are disordered in the electron density map.

The structure of SpLsm4N was also determined by the SAD method using the data obtained from a SeMet derivative crystal. The structure has been refined at a resolution of 2.2 Å to an R factor of 23.7% and R_free_ of 25.2% with good geometry. The final model covers residues 12–71 of every molecule in the AU. Residues 1–11 and 72–91 are not visible in the electron density map and assumed to be disordered. Attempts of crystallization of full length SpLsm4 failed due to the poor solubility and low yield of the full length protein.

The crystal structure of SpLsm5/6/7 sub-complex was determined at a resolution of 2.3 Å by the SAD method, using phases derived from a SeMet derivative crystal. The model has been refined at the resolution of 2.3 Å to an R factor of 23.1% and R_free_ of 25.4% with good stereochemistry. Several regions are disordered, namely residues 1–5 and 78–80 in Sp-Lsm5, residues 74–75 in SpLsm6, and residues 1–31, 69–77 and 101–113 in SpLsm7. The statistics of data collection and refinement are summarized in **Table 1**.

**Table 1:** Data collection and refinement statistics.

	SpLsm3	SpLsm4N	SpLsm5/6/7
Data collection statistics			
Derivative	SeMet	SeMet	SeMet
Number of Se sites	28	48	8
Space group	P2_1_2_1_2_1_	C2	P4_1_2_1_2
Unit cell dimensions			
a/b/c (Å)	101.4, 101.7,143.4	185.1, 124.5, 131.6	69.4, 69.4, 172.3
α/β/γ (°)	90.0, 90.0, 90.0	90.0, 135.0, 90.0	90.0, 90.0, 90.0
Wavelength (Å)	0.9795	0.9795	0.9792
Resolution limit (Å)	2.7	2.2	2.3
Completeness (%)^a^	99.6 (99.9)	99.3 (91.6)	96.3 (80.5)
Rmerge (%)^a^	7.1 (41.7)	6.1 (20.8)	9.7 (54.2)
*<I/σ(I)>* a	9.4 (2.3)	13.5 (4.5)	10.9 (2.0)
Refinement statistics			
Resolution range (Å)	64.2–2.7	92.6–2.2	54.0–2.3
Used reflections (N)	41387	105147	18409
No. of molecules/ASU	14	24	3
*R_work_/R_free_* (%)^b^	24.3/27.7	23.7/25.3	23.1/25.4
No. of atoms			
Protein/water	8092/144	11927/1063	1579/71
Mean *B* value			
Protein/water	41.6/39.0	29.0/29.5	37.6/37.8
Root mean square deviations			
Bond length (Å)/Bond angle (degrees)	0.01/1.248	0.01/1.308	0.009/1.247
Ramachandran plot (%)^c^	87.0/12.8/0.2/0	90.4/9.6/0/0	89.6/10.4/0/0

a Values in the highest resolution shell are shown in parentheses.

b *R_work_*  = *Σ||Fobs| - |Fcalc||/Σ|Fobs|*. *R_free_* is calculated identically with 5% of randomly chosen reflections omitted from the refinement.

c Fractions of residues in most favoured/allowed/generously allowed/disallowed regions of the Ramachandran plot were calculated according to PROCHECK.

### Overall Architecture

**Figure 1:**
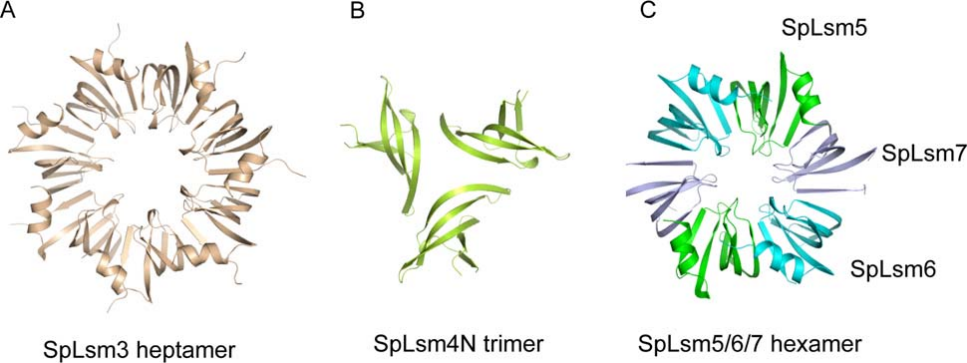
Ring structures of SpLsm3, SpLsm4N and SpLsm5/6/7 within crystal lattices. Structures are viewed from the helix faces of each ring structure. (A) The asymmetric unit of orthorhombic SpLsm3 crystal consists of 14 protein subunits, which are packed into two heptamers coaxially via helix face-helix face region. One SpLsm3 heptamer is shown. (B) The asymmetric unit of monoclinic SpLsm4N crystal contains 24 protein subunits, which are arranged as 8 copies of trimer. One SpLsm4N trimer is shown. (C) The asymmetric unit of tetragonal SpLsm5/6/7 crystal contains one copy of each subunit. Through symmetry operation, a closed hexamer ring structure is generated.

**Figure 2:**
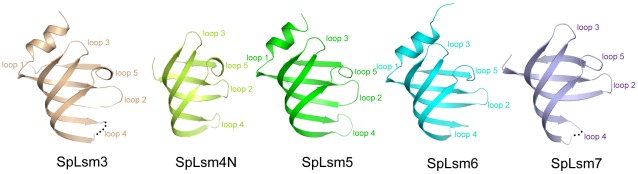
Overall architectures of SpLsm3, SpLsm4N, SpLsm5, SpLsm6 and SpLsm7. The monomeric structures of SpLsm3, SpLsm4N, SpLsm5, SpLsm6 and SpLsm7 are shown in cartoon with similar orientations. Each monomer is colored as in Figure 1. The disordered loop 4 region in SpLsm3 and SpLsm7 is shown as dotted lines.

SpLsm3 was crystallized with 14 copies of molecules in the AU, which packed into two heptamers coaxially via helix face-helix face region (**Fig. 1A**). Like its *S. cerevisiae* counterpart, ScLsm3 [33], each SpLsm3 subunit is made up of the N-terminal α helix (residues 10–17), followed by a highly curved five-stranded β sheet (β1, residues 19–26; β2, residues 30–40; β3, residues 43–54; β4, residues 71–81; β5, residues 86–89) (**Fig. 2**).

Within the AU of the SpLsm4N crystal, 24 molecules of SpLsm4N are arranged loosely as 8 copies of trimer (**Fig. 1B**). Each SpLsm4N molecule consists of a twisted β-sheet formed by five anti-parallel strands (β1, residues 14–19; β2, residues 22–33; β3, residues 36–47; β4, residues 50–61; β5, residues 67–70) while the α helix supposed to precede the β-sheet is disordered which is not due to the crystal packing after examination of the crystal lattice (**Fig. 2**).

Unlike SpLsm3 and SpLsm4N, the AU of the SpLsm5/6/7 crystal contains one copy of the trimeric complex. However, through symmetry operation, a closed hexameric ring can be generated within the crystal lattice (**Fig. 1C**), in which the two trimeric complexes are related by a crystallographic two-fold symmetry. In this hexamer, each subunit of SpLsm5 and SpLsm6 is composed of the N-terminal α helix (residues 7–14 of SpLsm5 and residues 4–12 of SpLsm6), capping the twisted five-stranded β sheet (**Fig. 2**) while the SpLsm7 subunit just contains the five-stranded β sheet without the N-terminal α helix. The five β-strands comprise residues 16–23 (β1), residues 27–37 (β2), residues 40–51 (β3), residues 55–66 (β4), residues 71–75 (β5) in SpLsm5, residues 14–21 (β1), residues 24–35 (β2), residues 38–49 (β3), residues 52–63 (β4), residues 68–72 (β5) in SpLsm6 and residues 33–40 (β1), residues 43–54 (β2), residues 57–67 (β3), residues 80–89 (β4), residues 93–98 (β5) in SpLsm7.

The structures of the five Lsm proteins described above indicate that these Lsm proteins have a common Sm fold. Superposition of the individual subunit of these Lsm proteins shows that the best match is located in the β-sheet region that comprises the two well conserved Sm motifs, with the variable loop 4 between β3 and β4 showing the largest structural deviation. As expected, each of the five Lsm proteins also shows high structural similarity to the human Sm proteins as well as to the bacterial and archaeal Lsm proteins as evidenced by the structural superpositions with the root mean square deviations (r.m.s.ds) over backbone Cα atoms ranging from 1.0 to 1.8 Å. Altogether, these results indicate the strict conservation of the Sm fold across the three kingdoms of life.

### Oligomeric States of SpLsm3, SpLsm5/6/7 and SpLsm4N

**Figure 3:**
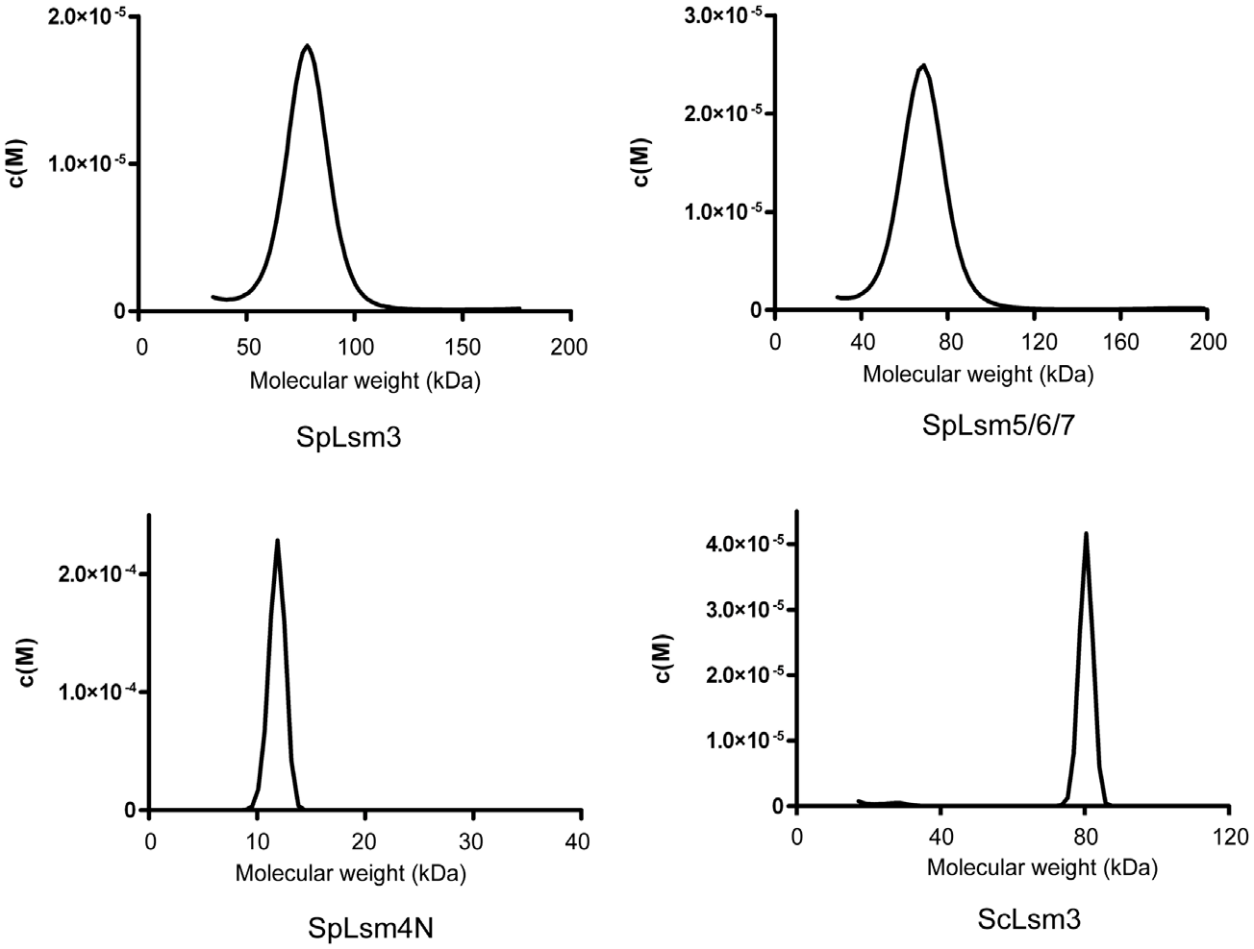
Sedimentation velocity study of Lsm proteins in solution at 0.75 mg/ml. The Lsm proteins including SpLsm3, SpLsm5/6/7, SpLsm4N and ScLsm3 were analyzed by sedimentation velocity and fitted based on the c(M) and c(S) size-distribution functions. The corresponding molecular weights obtained from the c(M) size-distribution function for SpLsm3, SpLsm5/6/7, SpLsm4N and ScLsm3 were 75.0 kD, 62.6 kD, 80.3 kD and 11.8 kD, respectively.

One of the hallmarks of the Lsm proteins is the propensity to form an oligomeric ring-like structure [32]. Consistent with this notion, our structures showed that a possible arrangement of a dimer of heptamers, a trimer and a hexamer for SpLsm3, SpLsm4N and SpLsm5/6/7, respectively, in the crystal lattice. To examine whether these oligomeric states also exist in solution, sedimentation velocity analysis of analytical ultracentrifugation (AUC) was employed using three different protein concentrations. The data were fitted by the continuous c(S) and c(M) distributions and gave the average molecular weights of 77.7 kD (SpLsm3) and 62.7 kD (SpLsm5/6/7), which are close to the theoretical molecular weights of 77.6 kD for homo-heptameric SpLsm3 and 62.5 kD for hetero-hexameric SpLsm5/6/7 (**Fig. 3** **,** **Table 2** **and** **3**). These results indicate that SpLsm5/6/7 forms a hetero-hexamer both in crystal and in solution while SpLsm3 is in a heptameric state. A dimer of heptamers for SpLsm3 observed in the crystal lattices is apparently induced by the crystal packing. Unlike SpLsm3 and SpLsm5/6/7 that show constant oligomeric state under different concentrations, a clear concentration dependent pattern was observed in SpLsm4N with the molecular weight ranging from 11.8 kD at low concentration to 22.9 kD at high concentration (**Fig. 3** **,** **Table 2** **and** **3**), suggesting that there exists a self association and dissociation equilibrium between the monomeric and oligomeric states. Sedimentation equilibrium analysis was then employed. Monomer and trimer model was found to fit well and gave the association constant value of 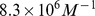
(**Figure S1**).

**Table 2:** Details of sedimentation velocity data analysis.

Protein	Concentration(mg/ml)	S (from c(S))	S_20,W_ a	f/f_0_	RMSD^b^	MW^c^ from c(M)
SpLsm3	0.75	4.24	4.58	1.40	0.01	75093
	1.0	4.32	4.67	1.40	0.05	77355
	1.5	4.46	4.82	1.39	0.04	80575
SpLsm5/6/7	0.75	4.00	4.24	1.33	0.01	62558
	1.0	4.07	4.31	1.33	0.05	63663
	1.5	4.09	4.34	1.30	0.01	61997
SpLsm4N	0.75	1.43	1.57	1.20	0.01	11823
	1.0	1.84	2.01	1.20	0.07	17231
	1.5	1.85	2.02	1.44	0.01	22894
ScLsm3	0.75	4.55	4.79	1.40	0.01	80271
	1.0	4.99	5.25	1.35	0.07	82518
	1.5	4.57	4.81	1.40	0.02	80798

a S20, w is the sedimentation coefficient with the parameter being corrected to 20.0°C and the density of water.

b RMSD is the root mean square deviation from SEDFIT program fitting.

c MW is molecular weight in Dalton.

**Table 3:** Oligomeric state of studied Lsm proteins determined from three different concentrations.

Protein	MW^a^ of monomer	Averaged MW (RMSD)	Oligomer
SpLsm3	11087	77674 (2755)	Heptamer
SpLsm5/6/7	31240	62739 (848)	Dimer
SpLsm4N	12204	17316 (5536)	Not determined
ScLsm3	10450	81196 (1175)	Octamer

a MW is molecular weight in Dalton.

**Figure 4:**
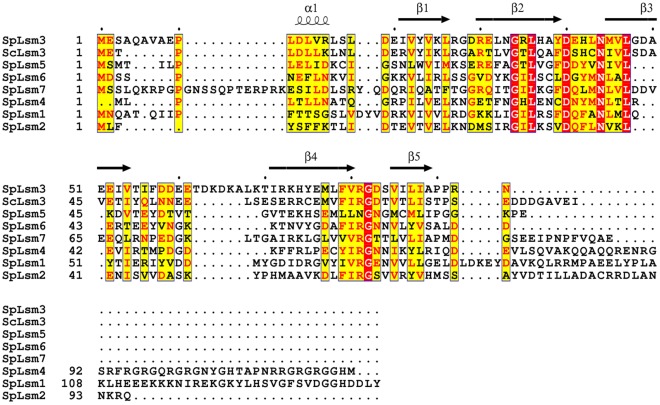
Sequence alignment of Lsm1 to Lsm7 proteins from *S. pombe* (Sp) and Lsm3 from *S. cerevisiae* (Sc). The secondary structural elements of SpLsm3 are shown on top of the sequences.

In contrast to the heptamer formed by SpLsm3, the crystal structure of ScLsm3 showed that it forms two coaxially packed and helix-to-helix faced octamers in the crystal lattice [33]. To validate the oligomeric state of ScLsm3 observed in the crystal, we used AUC to check whether ScLsm3 is in a heptameric or octameric state in solution using three different protein concentrations. Sedimentation velocity analysis gave a single peak, corresponding to the average molecular weight of 81.2 kD, which is close to the theoretical value of 83.6 kD for an octameric ScLsm3 (**Fig. 3** **,** **Table 2** **and** **3**). This indicates that ScLsm3 tends to form an octamer while SpLsm3 has the propensity to form a heptamer although these proteins share high sequence homology (**Fig. 4**).

### Surface Properties

The hexameric SpLsm5/6/7 and heptameric SpLsm3 ring structures are doughnut-shaped and formed by a continuous anti-parallel β sheet, wherein each subunit binds to its adjacent subunit via β-strand pairing between β4 and β5 (**Fig. 1**). The loops in each Sm motif (loops 2 and 3 in Sm motif 1 and loop 5 in Sm motif 2) form the inner surface of the ring structure while the helix in Sm motif 1 and loop 4 connecting the two Sm motifs constitute the two faces of the ring, i.e the helix face and loop face, respectively.

The hexameric SpLsm5/6/7 ring has an outer diameter of 57.0 Å, an inner diameter of 10.8 Å and a thickness of 32.0 Å, as compared to 61.5 Å of outer diameter, 20.7 Å of inner diameter and 31.0 Å of thickness for the SpLsm3 heptameric ring (**Fig. 1**). The thickness of the SpLsm3 heptamer is probably underestimated as loop 4 of SpLsm3 is disordered.

**Figure 5:**
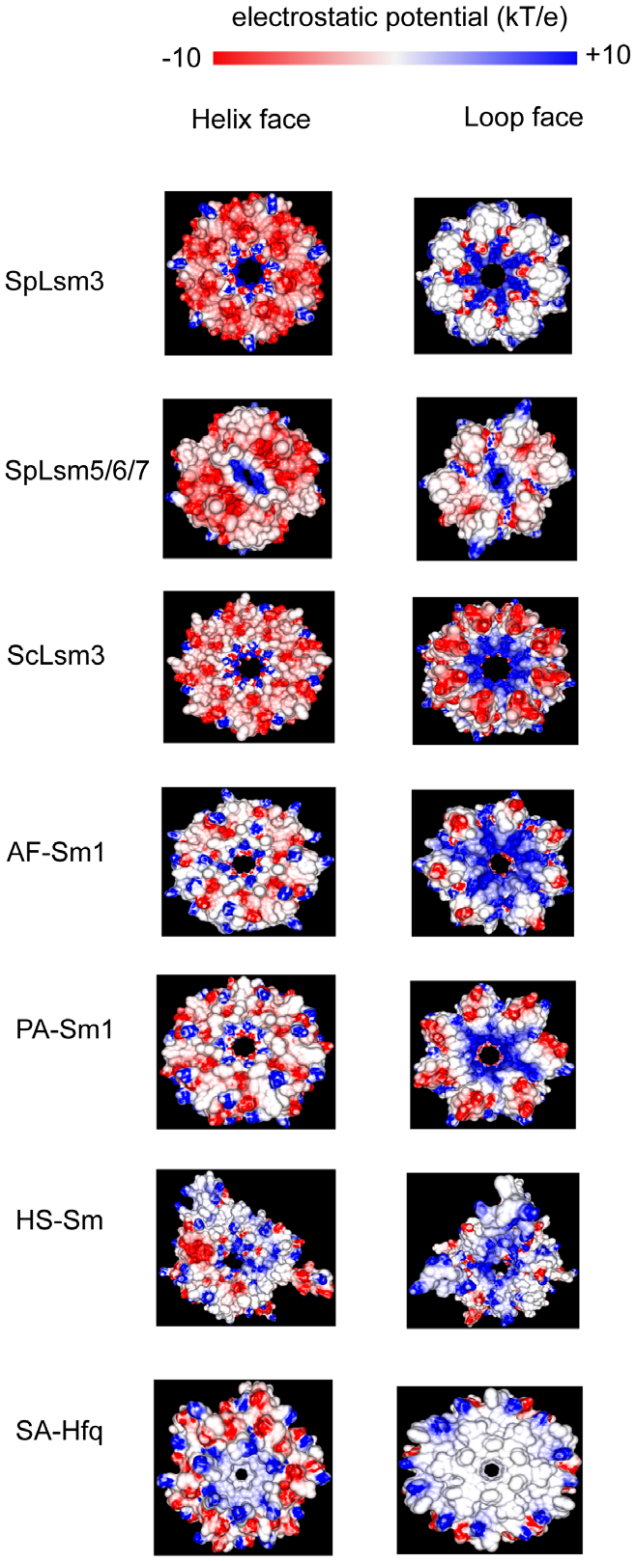
Electrostatic potential of Lsm and Sm proteins viewed from the helix and loop faces. *Archaeoglobus fulgidus* Sm1 protein (AF-Sm1) (PDB code 1I4K); *Pyrococcus abyssi* Sm1 (PA-Sm1) (PDB code 1M8V); *Homo sapiens* Sm complex (HS-Sm) (PDB code 2Y9A); *Staphylococcus aureus* Hfq (SA-Hfq) (PDB code 1KQ1). The figure was generated with GRASP2.

Electrostatic potential mapping on the molecular surface of SpLsm5/6/7 revealed different charge distribution patterns on its helix and loop faces (**Fig. 5**). Prominent negatively charged patches dominate the helix face of SpLsm5/6/7 while neutral charge is prevalent on the loop face. Moreover, the hexameric SpLsm5/6/7 lacks a 6-fold symmetry; therefore the SpLsm5/6/7 ring is not a real hexamer and may be best described as a dimer of trimers. Such organization of SpLsm5/6/7 may be important in its assembly with other Lsm proteins or RNAs to form more complicated complexes such as Lsm1–7 or in complex with RNAs.

Like SpLsm5/6/7, mapping of the electrostatic potential on the surface of SpLsm3 revealed distinct charge distribution patterns on its helix and loop faces (**Fig. 5**). The helix face is predominantly negatively charged while a 7-blade turbine like positively charged patch emanates from the cavity with neutral charge regions surrounding the outer side of the loop face. Unlike the elliptical cavity in the ring of SpLsm5/6/7, the cavity in the SpLsm3 heptamer is round and a 7-fold symmetry can be clearly identified. ScLsm3 forms an octameric ring [33] instead of a heptameric ring. Like the SpLsm3 heptamer, the helix face of octameric ScLsm3 is pronounced with negatively charged patches while the outer region of its loop face shows distinctly different charge distribution from that of SpLsm3 (negative vs. neutral charge) (**Fig. 5**).

The crystal structures of several Sm/Lsm proteins in complex with RNA have been solved. These include Lsm proteins from archea, AF-Sm1 in complex with oligo (U) [28] and PA-Sm1 in complex with oligo (U) [30], bacterial SA-Hfq with bound oligo (U) [29] and human Sm core in complex with U1 and U4 snRNAs [5], [6]. Inspection of the electrostatic potential distribution on the surfaces of these ring structures (**Fig. 5**) reveals a relatively conserved charge distribution pattern in the helix face, i.e., a neutral charge dominant surface interspersed with the positively and negatively charged clusters. By comparison, the loop faces of these ring structures showed diverse charge distribution patterns. The U-rich tract of the RNA ligands have been shown to bind to the helix faces of these ring structures while the loop face of *E. coli* Hfq has been shown to interact with the oligo(A) tract [34]. Given the predominantly negatively charged surfaces of the helix faces of SpLsm3, ScLsm3 and SpLsm5/6/7, the U-rich RNA oligo may not be able to bind these faces. Consistently, Sobti and co-workers [35] showed that the ScLsm3 octamer has no detectable affinity with the RNAs containing U-tract.

### RNA Binding Properties of SpLsm2/3, SpLsm3, SpLsm5/6/7 and SpLsm4N

**Figure 6:**
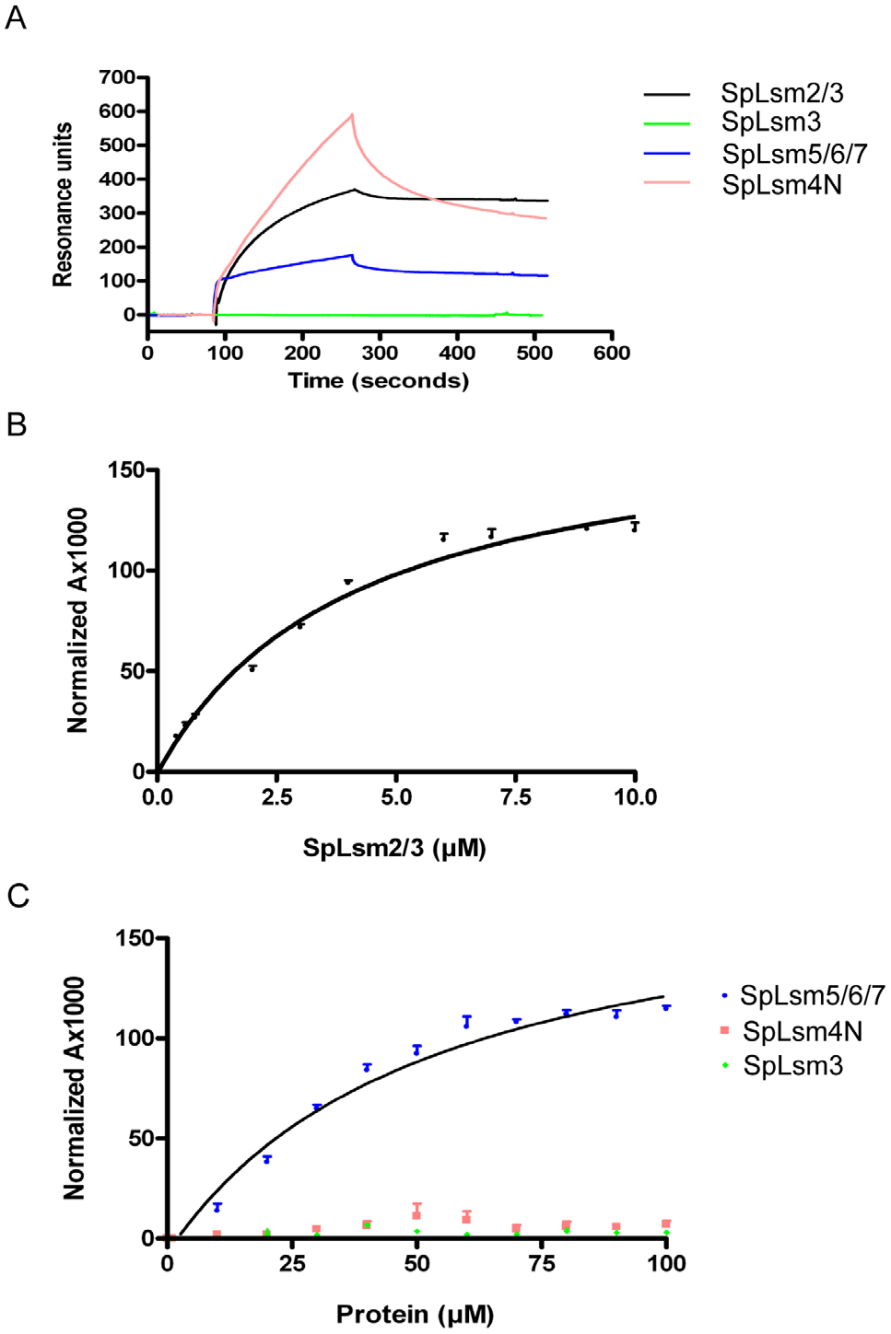
Analysis of U_15_ binding activity of SpLsm2/3, SpLsm3, SpLsm5/6/7 and SpLsm4N. (A) Sensorgrams of surface plasmon resonance analysis using 5′-end biotin-labeled U15. Fluorescence anisotropy analysis using 5′-end FAM-labeled U15 showed that the fitted K_d_ value of SpLsm2/3 is 4.0 ± 0.5 µM (B) and the fitted K_d_ value of SpLsm5/6/7 is 52.5 ± 10.0 µM while no K_d_ values could be determined for SpLsm3 and SpLsm4N (C).

To examine the RNA binding properties of SpLsm2/3, SpLsm3, SpLsm5/6/7 and SpLsm4N, surface plasmon resonance (SPR) analysis was used with 5′ biotin-labeled U_15_ attached to a streptavidin chip. The data from the SPR assays showed that SpLsm2/3 and SpLsm5/6/7 could interact with U_15_ whereas SpLsm3, like ScLsm3, failed to bind U_15_ (**Fig. 6A**), in agreement with the electrostatic potential mapping (see above). The distinct RNA binding properties were also observed in the case of ScLsm2/3 versus ScLsm3 [35]. Since the helix faces of the SpLsm3 heptamer and the ScLsm3 octamer are mainly negatively charged, the charge-charge repulsion would prevent the RNA from binding to the helix faces of these two complexes. The binding of the Lsm2 subunit to Lsm3 may change the charge distributions of Lsm3 by neutralizing its negatively charged potentials, therefore enabling the Lsm2/3 complex to bind the RNA oligos. Unlike the sensorgrams of SpLsm2/3 and SpLsm5/6/7, the sensorgram of SpLsm4N (**Fig. 6A**) revealed a fast-association and fast-dissociation pattern, which indicates the binding of SpLsm4N towards U_15_ is weak and transient.

The observation that SpLsm5/6/7 also binds to U_15_ contradicts with the electrostatic potential mapping on its surface as the negatively charged helix face would prevent RNA binding. The SpLsm5/6/7 hexamer is formed by two SpLsm5/6/7 trimers related by a 2-fold symmetry. Such an assembly of the SpLsm5/6/7 hexamer would allow the SpLsm5/6/7 trimer dissociated from the hexameric SpLsm5/6/7, thereby partially or fully exposing the positively charged central cavity to enable RNA binding. Alternatively, the RNA could bind to the loop face of this hexamer.

Fluorescence anisotropy analysis was performed to cross-check the U_15_ binding properties of SpLsm2/3, SpLsm3, SpLsm5/6/7 and SpLsm4N. In agreement with SPR analysis, SpLsm2/3 and SpLsm5/6/7 showed U_15_ binding affinities with the K_d_ values of 4.0 µM for SpLsm2/3 (**Fig. 6B**) and 52.5 µM for SpLsm5/6/7 (**Fig. 6C**) while the K_d_ value cannot be determined for SpLsm3 and SpLsm4N proteins (**Fig. 6C**) due to very weak RNA binding.

### Inter-subunit Contacts in SpLsm5/6/7

The interaction between strand β4 in one subunit and strand β5 in the adjacent subunit, which leads to the formation of a continuous anti-parallel β sheet in the ring-like structure, is a hallmark of all currently available ring structures of the Sm and Lsm proteins. In the subunit interface, in addition to the main chain-main chain hydrogen bonding interaction between β4 and β5, other interactions involving the side-chains of amino acids including ionic interactions and hydrophobic interactions also have been observed within the different oligomeric structures.

**Figure 7:**
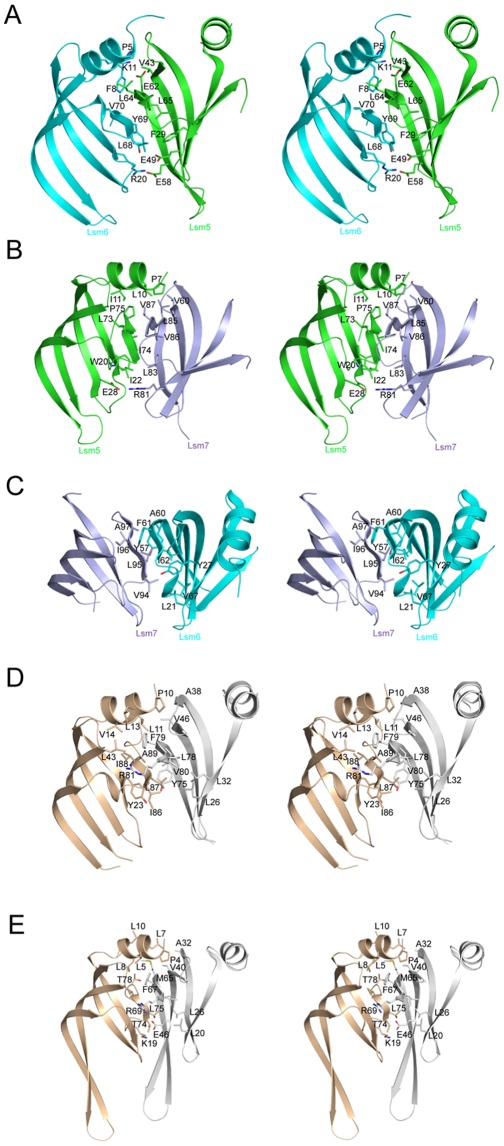
Subunit interfaces in SpLsm5/6/7, SpLsm3 and ScLsm3. Residues involved in interface interaction are shown in stick model. All subunit interfaces are shown in similar orientations. (A) Stereo view of the interface between SpLsm5 and SpLsm6. (B) Stereo view of the interface between SpLsm5 and SpLsm7. (C) Stereo view of the interface between SpLsm6 and SpLsm7. (D) Stereo view of subunit interfaces of SpLsm3. One subunit is colored as in Figure 1 while the other subunit is shown in grey. (E) Stereo view of subunit interfaces of ScLsm3 (PDB code 3BW1). The coloring scheme of the two subunits is as in Figure 7D.

Like all Sm/Lsm oligomeric assemblies, formation of the SpLsm5/6/7 hexamer is mediated through the interaction of β4 and β5 in two neighboring Lsm subunits. The hexameric SpLsm5/6/7 ring gives three possible types of inter-subunit contacts, namely the SpLsm5/6, SpLsm5/7 and SpLsm6/7 interfaces. In the SpLsm5/6 interface, β4 of SpLsm5 pairs with β5 of SpLsm6 to form an extended anti-parallel β sheet (**Fig. 7A**), which is further stabilized by two hydrophobic clusters and three salt bridges. The first hydrophobic cluster formed by Phe29 (β2), Leu64 and Leu65 (β4) of SpLsm5 and Leu68, Tyr69 and Val70 (β5) of SpLsm6 while the second one comprises Pro5 and Phe8 of the amphipathic helix in SpLsm6 and Val43 (β3) and Leu64 (β4) of SpLsm5. Lys11 from the amphipathic helix of SpLsm6 forms the first salt bridge with Glu62 (β4) of SpLsm5 on the helix-face side of the β sheet while Arg20 (β1) of SpLsm6 establishes two salt bridges with Glu49 (β3) and Glu58 (β4) of SpLsm5 on the loop-face side of the β sheet (**Fig. 7A**).

The interaction of SpLsm5 with SpLsm7 is similar to that of the SpLsm5/6 interface, which involves the pairing of β5 of SpLsm5 with β4 of SpLsm7 supplemented with hydrophobic clusters and salt bridges. One hydrophobic cluster involves Trp20, Ile22 (β1), Leu73, Ile74, Pro75 (β5) of SpLsm5 and Leu83, Leu85, Val86 and Val87 (β4) of SpLsm7 while the other hydrophobic core is formed by Pro7, Leu10, Ile11 (helix) of SpLsm5 and Val60 (β3), Leu85, Val87 (β4) of SpLsm7 (**Fig. 7B**). A salt bridge is formed between Glu28 (β2) of SpLsm5 and Arg81 (β4) of SpLsm7 on the loop-face side of β sheet (**Fig. 7B**) whereas no salt bridge is identified on the helix-face side of β sheet as compared to those observed in the SpLsm5/6 interface. The lack of this salt bridge is due to the presence of Leu85 in SpLsm7, which is equivalent to Glu-62 in SpLsm5 (**Fig. 7A and 7B**).

In the interface of SpLsm6 and SpLsm7, β5 of SpLsm7 interacts with β4 of SpLsm6 to form a continuous anti-parallel β sheet (**Fig. 7C**). However, no ionic interaction is observed in this interface, and only one hydrophobic cluster is identified, which involves Leu21 (β1), Tyr27 (β2), Tyr57, Ala60, Phe61, Ile62 (β4), and Val67 (loop 5) of SpLsm6 and Val94, Leu95, Ile96, Ala97 (β5) of SpLsm7 (**Fig. 7C**). The SpLsm6/7 interface buries a solvent-accessible surface of 1096 Å^2^ while the solvent-accessible surfaces of 1741 Å^2^ and 1777 Å^2^ are buried in the interfaces of SpLsm5/6 and SpLsm5/7, respectively. Recently, Mund and co-workers [36] solved the structure of SpLsm5/6/7 in a different crystal form and had a similar finding that the SpLsm6/7 interface has fewer contacts as compared to the interfaces of SpLsm5/7 and SpLsm5/6. These independent studies suggest that the interaction of SpLsm6 with SpLsm7 is weaker than those of SpLsm5/6 and SpLsm5/7 and thus SpLsm5 is most likely to bridge the interactions between SpLsm6 and SpLsm7 in the context of higher order ring structures such as Lsm1–7 and Lsm2–8.

### Inter-subunit Contacts in SpLsm3

In the SpLsm3 heptamer, the Lsm3 subunits are assembled one to another to form a heptameric ring via the interactions between strands β4 and β5′ (where ′ indicates the adjacent subunit) in two neighboring subunits. Specifically, Phe79 and Arg81 of β4 interact with Ile88 and Ile86 of β5′ respectively through main-chain hydrogen bonds (**Fig.** **7D**). Besides these hydrogen bonding interactions, extensive hydrophobic interactions are observed within the subunit interface which is composed of residues from α helix, β1, β3 and β5 of one subunit and β1′, β2′, β3′ and β4′ of the next subunit (**Fig. 7D**).

As mentioned above, SpLsm3 forms a heptamer while ScLsm3 exists as an octamer. Sequence alignment showed that these two proteins share 41% sequence identity (**Fig. 4**). Inspection of the subunit interfaces between the heptameric SpLsm3 and octameric ScLsm3 shows that they share the three conserved backbone hydrogen bonds between β4 of one subunit and β5′ of the adjacent subunit. However, two notable differences are found at the C-terminal region in both proteins (**Fig. 7D and 7E**). A backbone hydrogen bond is established between Met65 (β4) and Thr78 (C-terminal of β5′) in ScLsm3 (**Fig. 7E**) whereas such a hydrogen bond is not observed in SpLsm3 between the corresponding pair of Met77 (β4) and Pro90 (C-terminal of β5′), which is presumably due to the replacement of Thr78 in ScLsm3 by Pro90 in SpLsm3. The other difference is that a salt bridge is formed between Lys19 (β1) and Glu46 (β3′) of ScLsm3 while this ionic interaction is absent in SpLsm3 (**Fig. 7D and 7E**).

### Concluding Remarks

The study of the function of the Lsm complexes in eukaryotes has been hampered by the fact that the Lsm proteins tend to form stable homo- or hetero-multimeric sub-complexes, and generation of a functional complex in vitro requires reconstitution of Lsm1–7, Lsm2–8 and Lsm2–7 under denaturing conditions. The human Lsm1–7 and Lsm2–8 complex have been successfully reconstituted but no crystal structures are available for these two complexes, probably due to the difficulty in separation of Lsm1–7 and Lsm2–8 from these sub-complexes. Based on the structure of ScLsm3 and the sequence alignment between Lsm and Sm proteins, the models of Lsm1–7 and Lsm2–8 have been proposed, in which Lsm5 bridges the interaction between Lsm6 and Lsm7 [33]. Our structural model of SpLsm5/6/7 combined with the AUC analysis supports the ternary arrangement of Lsm5, 6, and 7 in this model and agrees with that reported by Mund and co-workers [36]. An important goal of future research will be to determine the structures of Lsm1–7 and Lsm2–8 both in apo form and in complex with RNA for understanding how these Lsm complexes are assembled and how they recognize their target RNAs.

## Materials and Methods

### Cloning, Expression and Purification

Reverse transcription-polymerase chain reaction (RT-PCR) was employed to amplify the genes encoding full length Lsm2, Lsm3, Lsm5, Lsm6 and Lsm7 and a C-terminal truncated Lsm4 (residues 1–91, designated as Lsm4N) from *S. pombe*. The *Lsm3* gene of *Saccharomyces cerevisiae* was amplified from *S. cerevisiae* genomic DNA. For co-expressing SpLsm2/3 and SpLsm5/6, the *Lsm2* and *Lsm5 genes* were inserted into the multiple cloning sites 1 (MCS1) of the pETDuet-1 vector (Novagen) with an N-terminal His_6_-tag fused to SpLsm2 and SpLsm5 while the *Lsm3* and *Lsm6* genes were inserted into the MCS2. The gene encoding SpLsm4N was cloned into the MCS1 of pETDuet-1 with an N-terminal His_6_-tag and the *Lsm7* gene was constructed into the MCS2 of the pACYCDuet-vector1 (Novagen). The *Lsm3* genes from both *S. pombe* and *S. cerevisiae* were inserted into the MCS1 of a modified pETDuet-1 vector with an N-terminal His_6_-tag followed by a PreScission protease cleavage site. All the constructs were verified by automated DNA sequencing.

*E. coli* B834 (DE3) cells harboring the pETDuet-1 vectors for expressing SpLsm3, SpLsm4N, SpLsm2/3 and Lsm3 from *S. cerevisiae* (ScLsm3) were grown at 37°C in Luria broth (LB) media. For co-expressing SpLsm5/6/7, the pETDuet-1 vector expressing SpLsm5/6 and the pACYCDuet-1 vector expressing SpLsm7 were co-transformed into the B834 (DE3) strain and grown in LB media containing ampicillin and chloramphenicol at 37°C. At OD_600_ of 0.6, cells were induced with 0.1 mM isopropylthio-β-galactoside (IPTG) and grown at 18°C for an additional 12 hours prior to harvest. Cell pellets of SpLsm3, SpLsm2/3, SpLsm5/6/7 and ScLsm3 were resuspended and sonicated in buffer A containing 20 mM Hepes pH 7.5, 200 mM NaCl, 2 mM β-mercaptoethanol and 5 mM imidazole. Cell pellets of SpLsm4N were resuspended and sonicated in buffer B containing 20 mM Tris pH 8.5, 500 mM NaCl, 2 mM β-mercaptoethanol and 5 mM imidazole. Cell debris was removed by centrifugation at 18,000 rpm at 4°C. The supernatant containing His_6_-tagged proteins was incubated with TALON Co^2+^ column (Clontech, Inc) pre-equilibrated with either buffer A or buffer B. The target proteins were eluted in either buffer A or buffer B containing 200 mM imidazole with the exception of SpLsm5/6/7 that was eluted with the buffer containing 15 mM imidazole. The eluted His_6_-tagged SpLsm3 and ScLsm3 were cleaved with PreScission protease at 4°C overnight. After desalting into buffer A without imidazole, the cleaved SpLsm3 and ScLsm3 were loaded into a second TALON Co^2+^ column to remove the cleaved His_6_-tag. The protein samples SpLsm3, SpLsm2/3, SpLsm5/6/7 and ScLsm3 were further purified by Superdex-200 26/60 column (Amersham Biosciences) in buffer C of 20 mM Hepes pH 7.5, 100 mM NaCl, 2 mM dithiothreitol (DTT) and SpLsm4N was further purified by Superdex-75 26/60 column in buffer D of 20 mM Tris pH 8.5, 200 mM NaCl, 2 mM DTT. All protein samples were concentrated to ∼10 mg/ml. Selenomethionine (SeMet)-substituted SpLsm2/3, SpLsm4N and SpLsm5/6/7 were expressed in a minimal medium containing 20 mg/l SeMet, and purified as above and concentrated to ∼10 mg/ml.

### Crystallization

Hanging drop vapor diffusion method was used to grow crystals in 1 ml of reservoir solution at 15°C. The crystals of SeMet-SpLsm2/3 were grown by mixing 1 µl of protein sample with 1 µl of 0.1 M Mes pH 6.5, 0.2 M MgCl_2_, 40% 2-methyl-2, 4-pentanediol (MPD). The crystals of SeMet-SpLsm4N were obtained by mixing 1 µl of protein sample with 1 µl of 0.1 M Mes pH6.5, 0.1 M NaCl, 12% polyethylene glycol 4000 (PEG 4000) while SeMet-SpLsm5/6/7 was crystallized by mixing 1 µl of protein sample with 0.1 M Mes pH6.5, 0.1 M MgCl_2_, 32% PEG 400. Crystals of SeMet-SpLsm2/3 and SeMet-SpLsm5/6/7 were directly frozen into liquid nitrogen while crystals of SeMet-SpLsm4N were transferred in serial steps to the mother liquor containing 30% PEG 400 before freezing in liquid nitrogen.

### Data Collection, Structure Determination and Refinement

Single-wavelength anomalous diffraction (SAD) data sets of SeMet-SpLsm3, SeMet-SpLsm4N and SeMet-SpLsm5/6/7 were collected at the peak of selenium K edge on the beamline ID23-1 (ESRF, Grenoble, France). All data sets were integrated with Mosflm and merged and scaled with Scala from the CCP4 suite [37]. Phases of the SeMet-SpLsm3, SeMet-SpLsm4N and SeMet-SpLsm5/6/7 data sets were initially calculated using the phasing module Autosol from PHENIX program package [38]. In total, selenium sites for initial phase calculation were 28, 48 and 8 for SpLsm3, SpLsm4N and SpLsm5/6/7, respectively. Density modification and automatic model building were then performed using the AutoBuild module of PHENIX program package [38]. More than 60% of residues were auto-traced into the experimental electron density maps of SpLsm3, SpLsm4N and SpLsm5/6/7. The remaining models were built manually with COOT [39]. All refinements were conducted with the refinement module phenix.refine of PHENIX program package [38]. The model quality was checked with the PROCHECK program [40]. Data collection and final refinement statistics are summarized in **Table 1** **.** Structural pictures were prepared in Pymol (www.pymol.org) and electrostatic potential diagrams were drawn in GRASP2 [41].

### Sedimentation Velocity

Sedimentation velocity experiments were carried out at 42000 r.p.m and 20°C using a ProteomeLab XL-A analytical ultracentrifuge (Beckman Coulter) in quartz cells fitted with double-sector centerpieces. Absorption measurements were made at 180 s interval at 280 nm until the boundaries reached the cell bottom. Prior to centrifugation, all samples including SpLsm3, SpLsm5/6/7 and ScLsm3 were dialyzed extensively into 20 mM Hepes pH 7.5, 100 mM NaCl while SpLsm4N was dialyzed extensively into 20 mM Hepes pH 7.5, 500 mM NaCl as the stability of SpLsm4N at 20°C was poor under low salt conditions. The concentration of all proteins samples was in 0.75 mg/ml which was measured using NanoDrop Spectrophotometer 1000 with molecular weight and extinction coefficient option. The theoretical molecular weight and extinction coefficient values of each sample were obtained from http://web.expasy.org/protparam. SEDFIT program (SEDFIT version 12.52, http://www.analyticalultracentrifugation.com) was used to calculate the protein partial specific volumes. The calculated protein partial specific volumes were 0.7399 for SpLsm3, 0.7352 for SpLsm5/6/7, 0.7225 for SpLsm4N and 0.7332 for ScLsm3. SEDNTERP program (Sednterp version 1.09, http://www.rasmb.bbri.org) was used to calculate the solvent density and viscosity. The solvent density and viscosity were 1.00391 and 0.01026 for SpLsm3, SpLsm5/6/7 and ScLsm3 samples and 1.02022 and 0.01063 for SpLsm4N. The continuous c(S) distribution and continuous c(M) distribution methods from SEDFIT program [42] were employed to analyze the data.

### Sedimentation Equilibrium

Sedimentation equilibrium experiment was performed using quartz cells fitted with 6-channel centerpieces in a ProteomeLab XL-A analytical ultracentrifuge at 20°C. SpLsm4N was dialyzed extensively into 20 mM Hepes pH 7.5, 500 mM NaCl. The sedimentation equilibrium runs were carried out at multiple speeds (15,000, 18,000, 25,000 rpm), multiple wavelengths (230, 250 and 280 nm) and multiple protein concentrations (0.3, 0.6, 0.9, 1.2, 1.5 mg/ml). The sample was run for 20 h at each speed plus an additional 2 h for the collection of scans. After the equilibrium scans, a high-speed centrifuge run at 42,000 rpm was done to determine the residual absorbance for setting initial baseline offset values. The data were fitted to a monomer-trimer model using the program HETEROANALYSIS [43].

### Surface Plasmon Resonance (SPR) Assay

SPR was performed on a Biacore 3000 instrument at 25°C. The 5′-end biotin-labeled single stranded RNA oligo U_15_ purchased from Dharmacon was attached to a streptavidin-coated sensor chip (Biacore). A buffer of 20 mM Hepes pH 7.5, 150 mM NaCl and 0.005% (v/v) Tween 20 was flowed through the chip until the baseline was stable. The biotin-labeled RNA was then attached to the flow cell 2 by injecting 20 µl of 100 nM RNA in 0.3 M NaCl at a flow rate of 5 µl/min. After immobilization, flow cell 2 and reference flow cell 1 were blocked with 100 µl of 1 mg/ml biotin at flow rate 5 µl/min. A binding buffer of 20 mM Hepes pH 7.5, 100 mM NaCl was flowed across flow cells 1 and 2 for the purpose of equilibration. Before injection, all samples including SpLsm2/3, SpLsm3, SpLsm4N, SpLsm5/6/7 and ScLsm3 were dialyzed extensively against the binding buffer. A total of 90 µl of 1 µM protein sample was injected across the chip at 30 µl/min. The data were analyzed using the software program BIAevaluation 3.1.

### Fluorescence Anisotropy Assay

Fluorescence anisotropy assay was measured in a total volume of 100 µl in 20 mM Hepes pH 7.5, 100 mM NaCl at 25°C. 5′-end 6-carboxy-fluorescein (6-FAM)-labeled single stranded RNA oligo U_15_ purchased from Metabion was used at 0.1 µM while SpLsm2/3 from the range 10 nM up to 10 µM and SpLsm3, SpLsm4N and SpLsm5/6/7 from the range 1µM up to 100 µM was added. Plates were read after an incubation period of 30 min at room temperature using a Safire II microplate reader (Tecan) in fluorescence polarization mode (excitation at 470 nm; emission at 535 nm; 3 reads) and its Magellan software (version 6.5). Anisotropy (A) was calculated using the formula 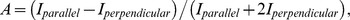 where *I_parallel_* and *I_perpendicular_* are the fluorescence intensities parallel and perpendicular to the excitation plane, respectively and a G factor of 1.08. Anisotropy values were normalized by subtracting the anisotropy in the absence of protein from all anisotropies and multiplied by 1000. Experiments were conducted in triplicate. Dissocation constants (K_d_) for protein and RNA interactions were calculated by nonlinear regression from each triplicate after normalization using Prism version 4 (GraphPad software) with the following equation:
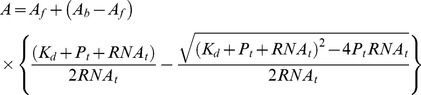



Where *A* is the anisotropy; A*_f_* and A*_b_* are the anisotropy values corresponding to free and bound RNA, respectively; and P*_t_* and RNA*_t_* are the total protein and RNA concentrations, respectively.

### Accession Numbers

The coordinates and structure-factor amplitudes for SpLsm3, SpLsm4N and SpLsm5/6/7 have been deposited in the Protein Data Bank with accession codes 4EMG, 4EMH, and 4EMK, respectively.

## Supporting Information

Figure S1 SpLsm4N was analyzed by sedimentation equilibrium and fitted to a monomer-trimer model. Representative fit was shown.
(TIF)
