# Supplementary figures and images for: Crystal Structures of Lsm3, Lsm4 and Lsm5/6/7 from Schizosaccharomyces pombe
Source: PLoS One. 2012 May 17;7(5):e36768. doi: 10.1371/journal.pone.0036768 (PMC3355152; doi:10.1371/journal.pone.0036768)

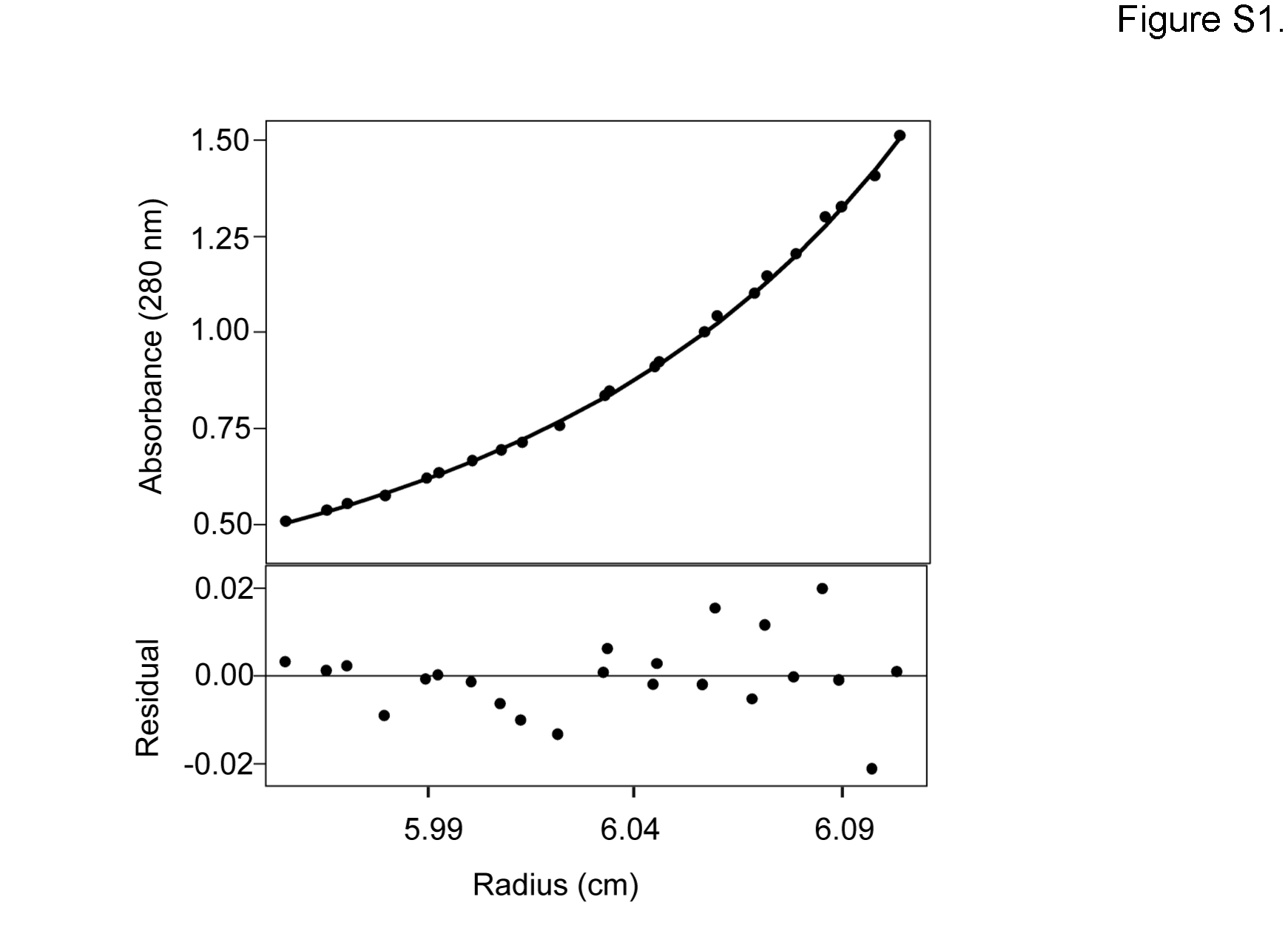

Supplement: Figure S1 — SpLsm4N was analyzed by sedimentation equilibrium and fitted to a monomer-trimer model. Representative fit was shown. (TIF) [file pone.0036768.s001.tif]
